# Characterising clinical *Staphylococcus aureus* isolates from the sinuses of patients with chronic rhinosinusitis

**DOI:** 10.1038/s41598-021-01297-0

**Published:** 2021-11-09

**Authors:** Brett Wagner Mackenzie, Melissa Zoing, Fiona Clow, David W. Waite, Fiona J. Radcliff, Michael W. Taylor, Kristi Biswas, Richard G. Douglas

**Affiliations:** 1 Department of Surgery, University of Auckland, Auckland, New Zealand; 2 Department of Molecular Medicine and Pathology, University of Auckland, Auckland, New Zealand; 3 School of Biological Sciences, University of Auckland, Auckland, New Zealand

**Keywords:** Microbiology, Molecular biology, Medical research

## Abstract

The role of *Staphylococcus aureus* in the pathogenesis of the chronic sinonasal disease chronic rhinosinusitis (CRS), has not been definitively established. Comparative analyses of *S. aureus* isolates from CRS with those from control participants may offer insight into a possible pathogenic link between this organism and CRS. The intra- and inter-subject *S. aureus* strain-level diversity in the sinuses of patients with and without CRS were compared in this cross-sectional study. In total, 100 patients (CRS = 64, control = 36) were screened for *S. aureus* carriage. The overall carriage prevalence of *S. aureus* in this cohort was 24% (CRS n = 13, control n = 11). Cultured *S. aureus* isolates from 18 participants were strain-typed using *spa* gene sequencing. The bacterial community composition of the middle meatus was assessed using amplicon sequencing targeting the V3V4 hypervariable region of the bacterial 16S rRNA gene. *S. aureus* isolates cultured from patients were grown in co-culture with the commensal bacterium *Dolosigranulum pigrum* and characterised. All participants harboured a single *S. aureus* strain and no trend in disease-specific strain-level diversity was observed. Bacterial community analyses revealed a significant negative correlation in the relative abundances of *S. aureus* and *D. pigrum* sequences, suggesting an antagonistic interaction between these organisms. Co-cultivation experiments with these bacteria, however, did not confirm this interaction in vitro. We saw no significant associations of CRS disease with *S. aureus* strain types. The functional role that *S. aureus* occupies in CRS likely depends on other factors such as variations in gene expression and interactions with other members of the sinus bacterial community.

## Introduction

*Staphylococcus aureus* is a commensal bacterium that colonises the human anterior nares, usually asymptomatically. However, *S. aureus* has increasingly become a focus of research interest because it possesses multiple virulence factors that can promote staphylococcal-related infection, as well as a range of antibiotic resistance genes^1–4^. Despite a substantial body of research highlighting the invasive nature of *S. aureus*, no consensus has been reached regarding change in *S. aureus* presence or abundance in patients with chronic sinonasal inflammatory disease, chronic rhinosinusitis (CRS)^5–8^. Determining carriage prevalence, number and types of strains in CRS patients would enhance understanding of the role of *S. aureus* in at least that subset of patients colonised by this putative sinonasal pathogen.

A plethora of host, bacterial and environmental risk factors affect susceptibility to *S. aureus* carriage and colonisation^9–11^. For example, the host produces a wide array of antimicrobial compounds such as peptides, antibacterial fatty acids and anti-staphylococcal lactoferrin, intended to protect against *S. aureus* colonisation. However, both commensal and virulent strains of this bacterium possess an arsenal of genes to combat the defences of the host^12^. These genes encode adhesive molecules, immunomodulatory factors, and a range of enzymes to circumvent defensive measures and enable nasal colonisation^13–15^.

Environmental factors, such as other members of the bacterial community, also influence *S. aureus* colonisation. Mutual exclusion from other commensal bacteria in the sinuses can restrict *S. aureus* carriage and colonisation^16–20^. An inverse relationship between *S. aureus* and *S. epidermidis*, *Streptococcus* species, *Dolosigranulum pigrum*, *Finegoldia magna*, *Propionibacterium acnes*, *Corynebacterium tuberculostearicum*, and various members of the phylum *Actinobacteria*, have been noted^19,21–24^. The relationship between *Corynebacterium* species and *S. aureus* is less clear, and contrasting results have been published^17,25–27^.

In this cross-sectional study we applied a range of cultivation and sequencing techniques to assess strain-level variability of *S. aureus* within and between patients, bacterial community composition, and in vitro interactions of *S. aureus* with the commensal bacterium *Dolosigranulum pigrum*. The following questions were specifically addressed: (1) how many strains of *S. aureus* are typically found in the middle meatus of patients with and without CRS? (2) Are certain *S. aureus* strains associated with CRS? (3) Are there bacterial community-level differences between CRS patients and disease control subjects that are culture positive for *S. aureus* carriage? (4) Do *S. aureus* isolates from CRS patients respond differently to control-patient derived isolates in co-cultivation experiments with the commensal bacterium *D. pigrum?*

## Results

A total of 100 patients (CRS = 64, controls = 36) were swabbed over the course of the sampling period. Of these, 24/100 were *S. aureus* culture-positive, and data from these 24 patients were used in demographic and carriage analyses. *S. aureus* isolates were collected from 18/24 participants for strain-type analyses using *spa* gene sequencing and co-culture experiments. Additional swab samples were collected from the middle meatuses of these 18 participants for bacterial community composition analyses using amplicon sequencing of the V3V4 hypervariable region of the bacterial 16S rRNA gene.

### Demographic analyses and carriage prevalence of study population

Control (n = 36) and CRS patients without and with polyposis (CRSsNP and CRSwNP, respectively) were screened for *S. aureus* carriage at the time of surgery (CRSsNP n = 27, CRSwNP n = 37) (Table 1). No significant differences were detected between cohorts in age (control = 44 years ± 16 (mean ± SD); CRSsNP = 45 ± 16; CRSwNP = 48 ± 16) (Kruskal–Wallis test, *p* > 0.05). Overall, more males exhibited CRSwNP than females (26 males versus 11 females, *p* = 0.02); however, no other significant differences between sex and diagnosis were observed (control = 21/36 female, CRSsNP = 10/27, *p* > 0.05 for both). A majority of patients had never smoked (83/100) and most patients were of New Zealand European ethnicity (84/100). The average Lund–Mackay score, a radiologic score used to assess CRS severity, for CRSwNP patients (17.1 ± 4.9) was significantly higher than for CRSsNP patients (13.8 ± 2.5) (Mann–Whitney *U* test, *p* < 0.05).

**Table 1:** Demographic and clinical characteristics on the full cohort of sampled participants in this study (n = 100). *CRSsNP* chronic rhinosinusitis without nasal polyposis, *CRSwNP* chronic rhinosinusitis with polyposis.

Variable	Disease control (n = 36)	CRSsNP (n = 27)	CRSwNP (n = 37)	Unadjusted *p*-value
Positive *Staphylococcus aureus* carriage	11	7	6	N.S.
Age (years)	44 ± 16	45 ± 16	48 ± 15	N.S.
Gender (male)	15	17	26	*p* < 0.05
New Zealand European	29	25	30	N.S.
Smoker and ex-smoker	7	4	5	N.S.
Lund–Mackay score	–	14 ± 2.5	17 ± 4.8	*p* < 0.05

The overall carriage prevalence of *S. aureus* in this cohort was 24% (CRS n = 13, control n = 11). Carriage prevalence for CRSwNP (16.2%, n = 6) patients was lower when compared with CRSsNP (25.9%, n = 7) patients, however this difference was not significant (Mann–Whitney *U* test, *p* > 0.05). Although most *S. aureus* culture-positive disease control subjects were female (7:4, female:male), and a majority of CRS subjects were male (4:9), no significant association of sex to disease state was observed (Fisher’s exact test, *p* > 0.05).

Analysis of variance of Lund–Mackay radiologic severity scores and carriage status of CRS patients suggested *S. aureus* carriage was not associated with worse disease in CRSsNP or CRSwNP patients (Kruskal–Wallis with Dunn’s correction, *p* > 0.05). However, *S. aureus* culture-negative CRSwNP patients had significantly higher Lund–Mackay scores when compared to CRSsNP patients without *S. aureus* (Kruskal–Wallis with Dunn’s correction, *p* < 0.05). These results suggest that polyposis, rather than *S. aureus* carriage status, is associated with significantly more severe disease in CRS patients.

### Strain-level diversity of *S. aureus* in CRS patients and controls

Of the 24 patients that were *S. aureus* positive, isolates from 18 patients were collected and analysed (CRS = 10, disease control = 8). Almost half of these patients (7/18) had immune-related comorbidities such as asthma, cystic fibrosis, or Graves’ disease (Table S1). Furthermore, the majority of these 18 patients were prescribed antibiotics and steroids at the time of surgery (14/18), and many had extensive antibiotic and steroid prescription histories.

To investigate intra-personal strain-level diversity of *S. aureus* species in the middle meatus, multiple single colonies from the initial sample cultivation were collected from a subset of CRS patients and controls. Genotyping of the *S. aureus* isolates using the *spa* gene revealed that all six of these participants (three CRS and three controls) carried a single *spa*-type at the time of sampling (Table 2). Although these initial tests showed that diversity of *S. aureus* strain was monoclonal (only one *spa*-type was recovered in all subjects), up to five colonies from the initial cultivation agar were collected when possible for each subsequent *S. aureus*-positive subject.

**Table 2:** Characteristics of the *Staphylococcus aureus* isolates recovered in this study for chronic rhinosinusitis (n = 10) and disease control (n = 8) patients. *Staphylococcal* protein A (*spa*) typing results and reported relative global frequency distributions were determined using Ridom StaphType software v2.2.1. Isolates were considered methicillin-resistant *S. aureus* (MRSA) if they exhibited resistance to penicillin and flucloxacillin antibiotics. *CRS* chronic rhinosinusitis.

Subject	Diagnosis	#Colonies isolated	*spa-*type	Global frequency (%)	Antibiotic resistance
S01	Control	5	*t002*	5.93	Penicillin
S02	CRS	5	*t189*	0.43	Penicillin
S03	CRS	5	*t1451*	0.08	Penicillin, Erythromycin
S04	CRS	5	*t015*	1.19	Penicillin
S05	CRS	1	*t3258*	< 0.01	Penicillin
S06	CRS	1	*t015*	1.19	Penicillin
S07	CRS	20	*t084*	1.64	Penicillin
S08	CRS	20	*t732*	< 0.01%	Penicillin, Erythromycin
S09	CRS	20	*t528*	0.04	No
S10	Control	20	*t878*	0.02	Penicillin
S11	Control	20	*t127*	1.58	Penicillin
S12	Control	16	*t505*	0.04	Penicillin
S13	CRS	4	*t015*	1.19	Penicillin, Erythromycin
S14	CRS	5	*t189*	0.43	Penicillin
S15	Control	4	*t692*	0.04	Penicillin, Erythromycin, Flucloxacillin, Doxycycline (MRSA)
S16	Control	5	*t1346*	0.01	Penicillin
S17	Control	2	*t179*	0.07	No
S18	Control	2	*t015*	1.19	No

All participants in this study harboured monoclonal *spa*-types at the time of sampling. There was no observed skewing of *spa*-type with CRS status, as most *spa*-types were unique. The most frequently detected *spa*-types were t015 (n = 4) and t189 (n = 2). One methicillin-resistant *S. aureus* (MRSA) isolate was identified (t692).

### Bacterial community analyses

The V3V4 hypervariable regions of the bacterial 16S rRNA gene were amplified from samples originating from the 18 patients (10 CRS, 8 disease controls) that cultured positive for *S. aureus*. Sequences assigned to *S. aureus* were detected in all 18 patients. Permutational analysis of variance (adonis), based on 9,999 permutations and Bray–Curtis distances, revealed no significant contribution of disease status, polyposis, *spa*-type, or antibiotic resistances of *S. aureus* isolate to bacterial community composition (all *p* > 0.05). No significant differences between groups regarding alpha diversity or richness were detected (Supplementary Figure S1) and no distinct clustering in an nMDS was observed (Supplementary Figure S2). Specific differences in amplicon sequence variants (ASVs) between CRS patients and controls were explored in subsequent analyses.

**Figure 1:**
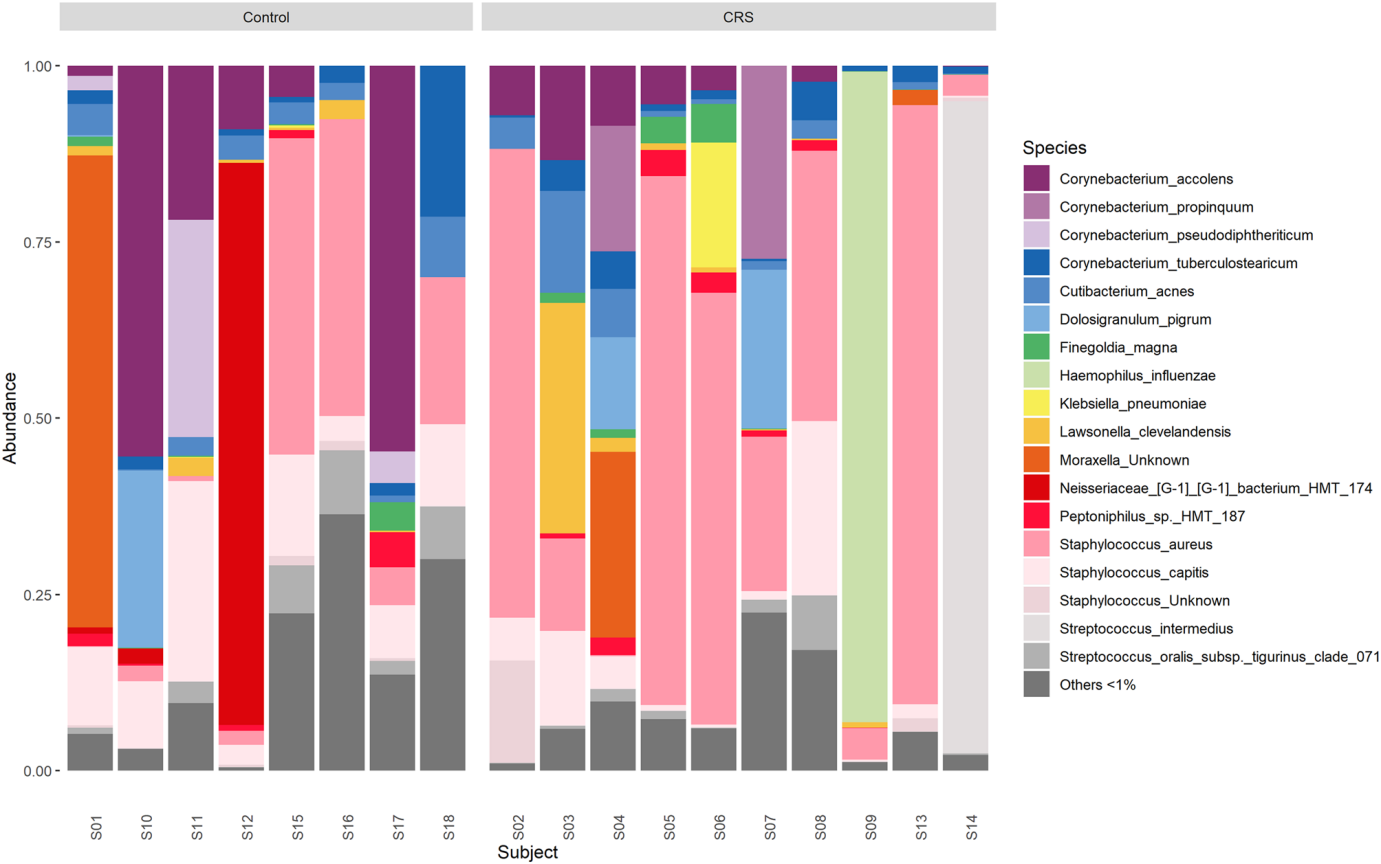
Bacterial community composition of taxonomy-assigned amplicon sequence variants summarized at the species-level in chronic rhinosinusitis (CRS, n = 10) and disease control (n = 8) patients that cultured positive for *Staphylococcus aureus*. The taxa bar plots display the 18 most abundant bacterial species with all others comprising < 1% of the relative abundance sequencing data grouped in ‘Others’. Taxa bar plots were generated in R statistical program version 3.6.1 and visualised using ‘ggplot2’ version 3.3.2.

The most abundant species were *S. aureus* (CRS = 36.9% ± 32.6% relative abundance of quality filtered sequence reads, disease control = 14.8% ± 18.9%), *Corynebacterium accolens* (CRS = 4.0 ± 4.6%, disease control = 18.4% ± 23.7%), and *Staphylococcus capitis* (CRS = 5.4% ± 7.9%, disease control = 11.1% ± 8.0%) (Fig. 1). A total of 8 ASVs were taxonomically assigned to *S. aureus*, of which ASV1 was the most abundant.

Differential abundance analyses of taxonomy-assigned ASVs revealed that ASV9 *Moraxella* (log_2_ fold change 24.4) and ASV41 *C. tuberculostearicum* (log_2_ fold change 20.8) were both significantly increased in CRS samples (“BH” corrected *p* < 0.001 for both). ASV1 *S. aureus* had greater proportions in CRS samples than controls, however after “BH” *p*-value adjustments this result was no longer significant. Other ASVs assigned to members of the genus *Corynebacterium*, namely ASV18 *C. accolens* (log_2_ fold change − 23.6) and ASV14 *C. pseudodiphtheriticum* (log_2_ fold change − 24.8), were significantly decreased in CRS patients (“BH” corrected *p* < 0.001 for both). ASV12 *Neisseriaceae* was also significantly decreased in CRS patients (log_2_ fold change − 24.9, “BH” corrected *p* < 0.001).

**Figure 2:**
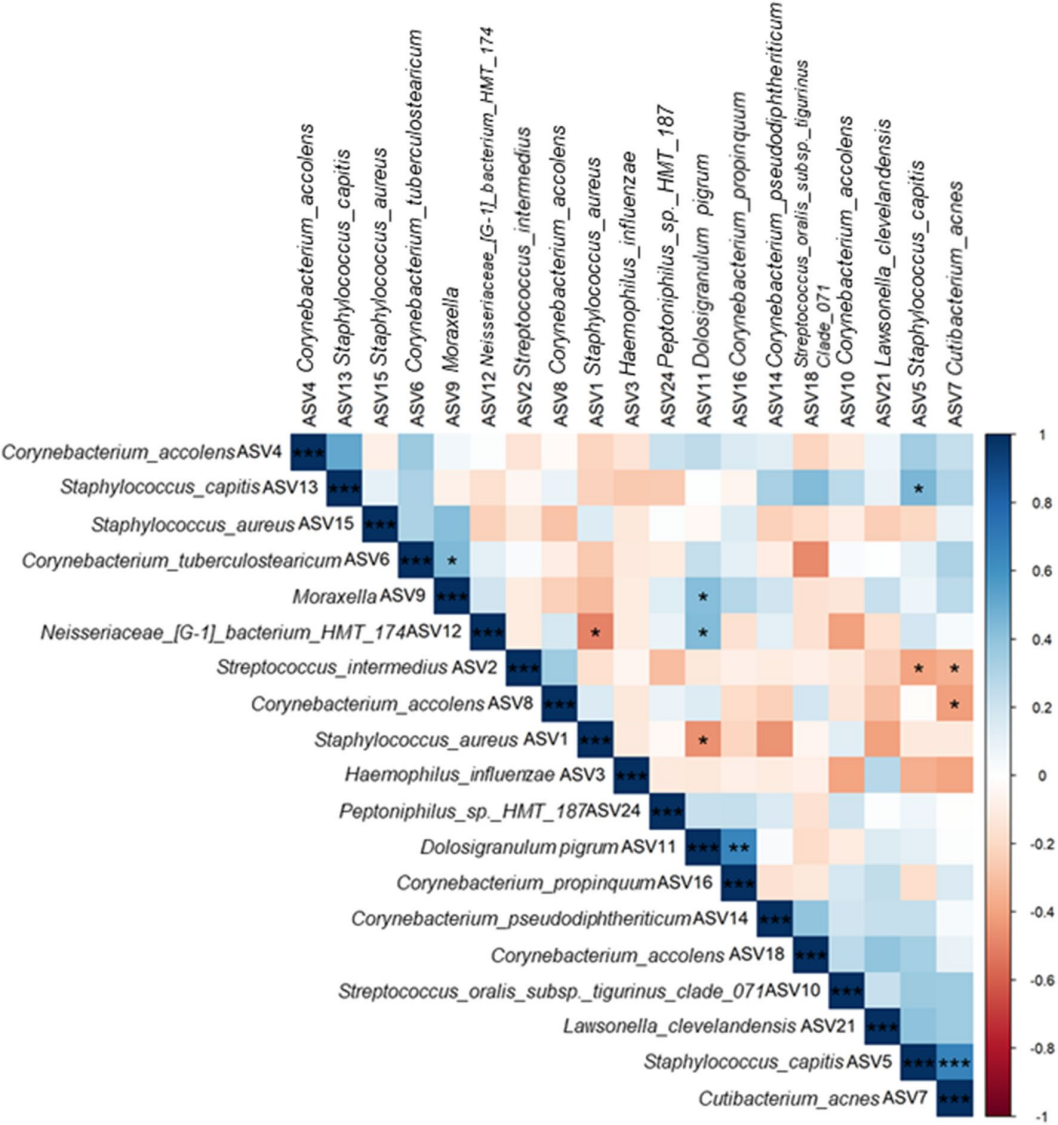
Spearman correlations displayed as a heatmap between taxonomy-assigned amplicon sequence variants (ASV) from bacterial community 16S rRNA gene amplicon sequence data. Both positive and negative correlations between bacterial ASVs were calculated from ASVs with more than 1% total relative abundance across all samples. Significance levels after “BH” *p*-value correction for multiple pairwise comparisons are displayed on the heatmap. ***p < 0.001, **p < 0.01, *p < 0.05. Spearman correlations were calculated in the R statistical program 3.6.1 package ‘corrplot’ and heatmaps were visualised using ‘ggplot2’ version 3.3.2.

Spearman correlations between ASVs with > 1% relative sequence abundances overall were investigated and revealed several significant positive correlations (Fig. 2). Notably, ASV5 *S. capitis* and ASV7 *Cutibacterium acnes*, and ASV11 *D. pigrum* and ASV16 *C. propinquum*, were significantly positively correlated. Fewer significant negative correlations were observed. Of importance, the most abundant ASV, ASV1 assigned as *S. aureus*, showed two significant negative associations with ASV12 *Neisseriaceae bacterium HMT174* and ASV11 *D. pigrum*.

### *S. aureus* and *D. pigrum* co-culture assay

**Figure 3:**
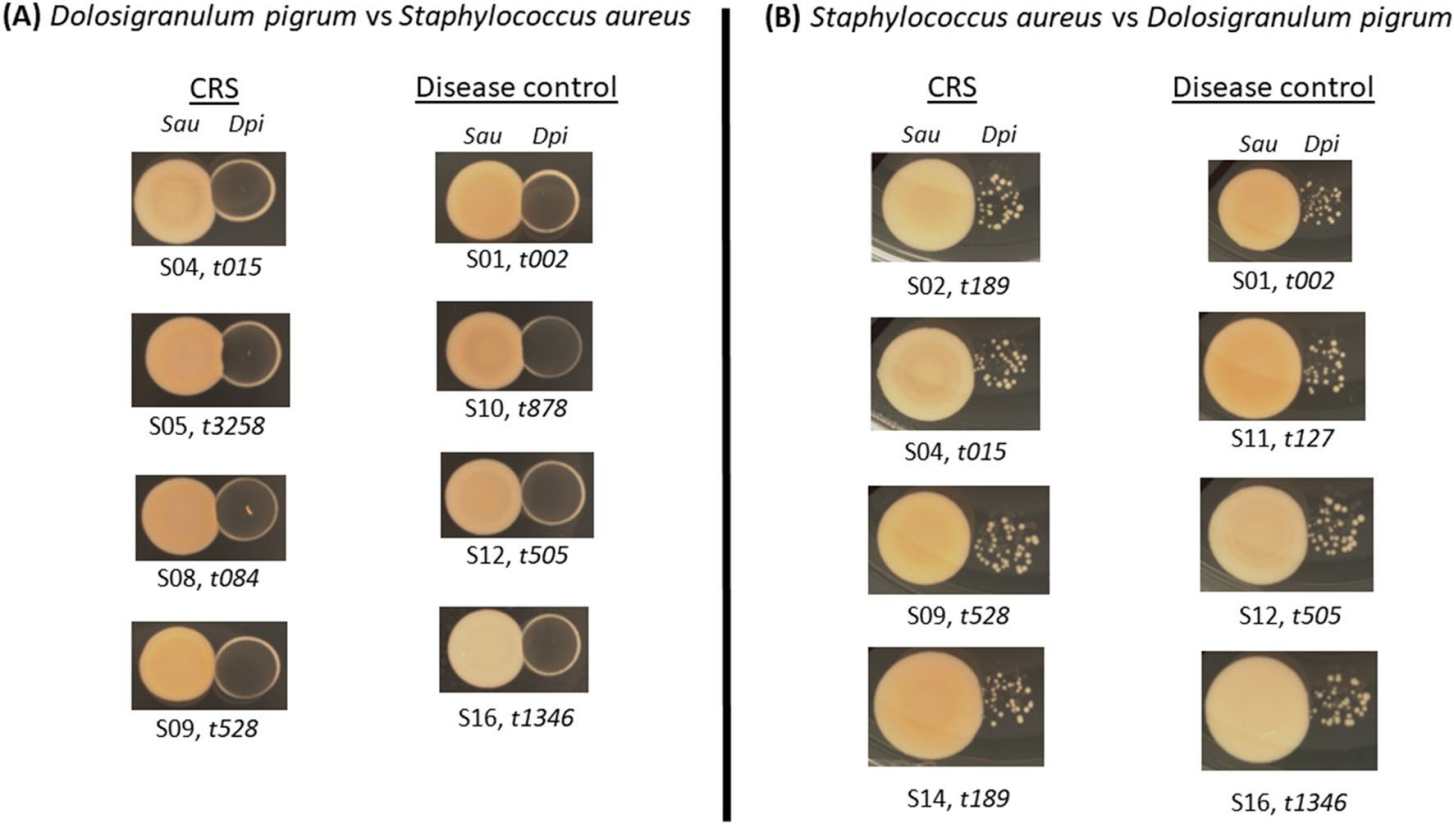
*Dolosigranulum pigrum* and *Staphylococcus aureus* co-culture assays on brain heart infusion (BHI) agar. Eighteen sinus-derived clinical isolates of *S. aureus* are (**A**) not inhibited by the pre-grown presence of *D. pigrum*, and (**B**) do not inhibit the growth of *D. pigrum*. Representative assays are shown for chronic rhinosinusitis and disease control *S. aureus* strains. *D. pigrum* was inoculated from BHI broth media onto BHI agar 2 days prior to inoculation of each *S. aureus* strain in (**A**). Each *S. aureus* strain was inoculated from BHI broth media onto BHI agar 1 day prior to inoculation with *D. pigrum* in (**B**). Co-culture assays were photographically documented 24 and 48 h after inoculation (48 h results are shown here for both **A** and **B**). Subject numbers and *S. aureus spa*-types are noted under each photograph.

The correlation results were used to guide co-cultivation assays. All *S. aureus* isolates were co-cultured with a single *D. pigrum* isolate obtained from the sinuses of a healthy volunteer with no history of CRS. Co-culture assays were assessed photographically as previously described (Brugger et al. 2019) and no antagonistic relationship between *D. pigrum* or any *S. aureus* strains was observed. The presence of established *D. pigrum* growth did not inhibit the subsequent growth of any of the clinical *S. aureus* strains, regardless of disease status (Fig. 3A). Similarly, the presence of any *S. aureus* strain prior to inoculation of *D. pigrum* did not impact on *D. pigrum* growth (Fig. 3B).

## Discussion

Studies focusing on the role of the microbiome in CRS have not reached a consensus regarding *S. aureus* presence, abundance, or strain-type associated with the disease^8,28–34^. Our study aimed to determine the intra- and inter-personal variability in *S. aureus* strain carriage and investigate *S. aureus* strain-type differences between CRS and disease control patients. The overall prevalence of nasal carriage of *S. aureus* in this cohort was 24%, which is slightly higher than previously observed local carriage rates^35^, but in line with global expected frequencies (20–30%)^11,36^. Very low levels of MRSA isolates were detected in our study, which is in keeping with previous results from an Auckland, New Zealand-based study that examined the dynamics of *S. aureus* carriage in healthy adults through time^37^.

*spa*-type analyses of *S. aureus* isolates did not reveal any trends in disease-specific strain-level diversity. All participants in this study harboured one strain type at the time of sampling, which suggests collection and strain-typing of a few isolates per person is sufficient to capture intra-personal cross-sectional diversity of *S. aureus* in the middle meatus. The results from this study are similar to other strain-typing studies which reported no differences in the distribution of strain-types or virulence gene prevalence in CRS and non-CRS participants^33,38,39^. More generally, the results from our study are consistent with a large scale, multi-centre European study which found that *spa*-type did not associate with invasive infection^65^.

The role of *S. aureus* in the aetiology of CRS is not well understood^39–42^. Previous evidence suggests that *S. aureus* is more common in patients diagnosed with CRSwNP; however, our study did not detect any significant differences between carriage prevalence in CRSsNP and CRSwNP patients^39,43^. The presence of polyposis, rather than *S. aureus* carriage, was associated with more severe disease. Our results agree with previous studies which suggest nasal colonisation of *S. aureus* is not associated with more severe disease^36^. Almost half of the participants in our study had immune-related comorbidities. It is not uncommon, however, for immune-compromised individuals to harbour *S. aureus* in their sinuses^4^. Additionally, many of the patients reported extensive antibiotic usage within the 12 months prior to sampling which may influence carriage status and bacterial community composition. A recent study examined the prescription patterns of antibiotics to CRS patients in comparison to disease controls and healthy participants in Auckland, New Zealand. This study found that the effects of previous antibiotic usage on the sinus bacterial composition were unpredictable^59^. It is important to note that such high antibiotic usage in our study may mask differences that would otherwise be detected between the CRS and disease control cohorts.

CRS has a multifactorial pathogenesis and heterogeneous clinical presentation. Recent research has applied statistical modelling to subgroup types of CRS disease using immunological or microbiological markers. One microbiological subgroup is characterised by an abundance of the bacterial family *Staphylococcaceae*, and another immunological subgroup is characterised by an increased abundance of staphylococcal enterotoxins^44,45^. A pressing need exists for examining each of these subgroups in isolation, especially the staphylococcal subgroup in light of its global importance as a major bacterial human pathogen, range of virulence factors and growing multi-drug resistance. Bacterial community analyses, in conjunction with cultivation and strain-typing of *S. aureus*, are more comprehensive than microbial amplicon sequencing analyses alone, allowing for improved predictions regarding the role of this bacterium in CRS disease.

Finer resolution of bacterial taxa achieved in this study by clustering of amplicon sequence variants (ASVs) and classification using the Human Oral Microbiome Database offered increased insight into bacterial community dynamics between species of interest in patients that are colonised by *S. aureus*. It is important to note, however, that identification of bacteria at the species-level should be interpreted with caution due to such short sequencing reads. Future studies should utilise full-length 16S rRNA gene amplicon sequencing in addition to quantitative PCR for species of interest. Co-occurring or competitive bacteria that have been studied previously include *Corynebacterium* and *Staphylococcus* species, *S. aureus* and *D. pigrum*, and *P. acnes* and *S. epidermidis*^19,22,24,25,46,47^. The results in our study suggest several other positive correlations may exist between *Peptoniphilus* and *Moraxella* sp., *C. propinquum*, and *Neisseriaceae* sp.

*Staphylococcus* species have well-documented competitive interactions^25,26,46^, and our bacterial community sequencing results suggested negative correlations between *S. aureus* and other bacteria. Sequencing data are compositional by nature and, therefore, differences observed according to relative sequence abundances may not accurately reflect in situ bacterial dynamics^48^. To this end, and although *D. pigrum* was not highly prevalent in patients (CRS = 2/10, disease control = 2/8), co-culture of *D. pigrum* with *S. aureus* was explored. Our co-cultivation results showed that the pre-grown presence of one sinus isolate of *D. pigrum* did not inhibit the subsequent growth of any *S. aureus* isolates. This contrasts with a recent study by Brugger and colleagues that showed 11 *D. pigrum* strains inhibited the growth of *S. aureus* strains JE2 and Newman^24^. Brugger and colleagues found that a diverse array of biosynthetic gene clusters may contribute to the inhibition of *S. aureus*. It could be that our *D. pigrum* and *S. aureus* isolates are genetically different from those used in the Brugger study. It is also important to highlight that the *D. pigrum* strain used in our co-cultivation studies was isolated from a healthy individual who was not *S. aureus* culture-positive and therefore excluded from this study. The *D. pigrum* strain could be different to that detected in the bacterial community sequencing results, and again to those *D. pigrum* strains tested by Brugger and colleagues. Future studies would benefit from including *S. aureus* and *D. pigrum* type strains as controls across studies.

Similar to the results by Brugger et al*.*, we observed that the pre-grown presence of any of the *S. aureus* strains did not inhibit the subsequent growth of *D. pigrum*. It could be that once *S. aureus* is established, a combination of factors is required to eradicate or inhibit the growth of this adaptable and virulent pathogen. Following on, the patients that cultured positive for *S. aureus* in our study may be persistent carriers of these strains, suggesting increased fitness compared with other commensal bacteria. Nonetheless, remediation of a dysbiotic sinonasal bacterial community (such as *Staphylococcus*, *Streptococcus*, and *Corynebacterium* subgroups in CRS) with competing bacterial strains remains a promising avenue for research, primarily as a therapeutic alternative to antibiotics or surgery in the treatment of CRS^49–53^.

Although our study is limited by cross-sectional sampling and small sample sizes, we combined culture and molecular techniques to provide a comprehensive analysis of the *spa*-type heterogeneity of *S. aureus* strains in CRS patients. We saw no significant associations of CRS disease with *S. aureus* strain types. The functional role that *S. aureus* occupies in CRS likely depends on other factors such as variations in gene expression and other members of the sinus bacterial community. Furthermore, some evidence suggests that *S. aureus* microcolonies residing in the intramucosal space more frequently in CRS patients than controls may be a contributing factor to the chronic nature of this disease^64^. Future studies cultivating *S. aureus* isolates should examine phenotypic differences between strains. Heterogeneity in haemolysis capability, small colony variant presentation, and differences in pigmentation offer insights into adaptations that could be related to pathogenic potential. Mechanisms underlying the relationship between the pathobiont *S. aureus* and the commensal bacterium *D. pigrum* should be explored further. Current research suggests that *S. aureus* is unlikely to be the sole causative agent of CRS, but it may play more of a pathogenic role in the subset of CRS patients that carry this bacterium. Future research should endeavour to include a healthy patient cohort not undergoing surgery and focus on whole genome sequencing of representative strains to identify genes of interest that may facilitate development of sinus disease.

## Materials and methods

### Patient information, sampling and culturing

Patients were recruited from Auckland City Hospital, New Zealand, commencing May 2015 and finishing September 2017 for this prospective, cross-sectional study. Patients were classified into one of two groups based on prevalence or absence of CRS disease. Disease control participants were undergoing surgery for reasons unrelated to CRS disease. Exclusion criteria were immunodeficiency and age < 18 years. Patient demographics, symptom severity scores assessed by Lund–Mackay computed tomography scoring^54^, and medical history (including co-morbidities and antibiotic usage) were collected from patients that cultured positive for *S. aureus* (Table S1). Patients with CRS were further classified according to presence or absence of nasal polyposis (CRSwNP or CRSsNP, respectively). Written informed consent from all patients in this study and ethical approval (NTX/08/12/126) from the New Zealand Health and Disability Ethics Committee were obtained. All experiments were performed in accordance with relevant guidelines and regulations. Paired sterile rayon-tipped swabs (Copan, #170KS01) from the left and right middle meatuses were collected for microbiological assessment at the time of induction of anaesthesia. Swabs were immediately placed in RNA*later* and transferred to the laboratory, where they were stored at − 20 °C until further analysis.

An additional sterile transport swab for bacterial culture (Transystem, Copan) was collected from the middle meatus side with the easiest access, to avoid contamination from other sinus sites. The mucosal swab samples were immediately placed in sterile transport medium and transferred to Auckland LabPLUS laboratories, located at Auckland City Hospital. Swabs were streaked onto blood agar plates and mannitol salt agar plates (MSA, *Staphylococcus-*selective media). Blood agar plates were incubated at 5% CO_2_, 37 °C and MSA plates were incubated at 37 °C, ambient oxygen concentration. Growth on plates was assessed after 18 h, and again after 48 h (from the time of plating, as per standard LabPLUS procedures).

### Identification of *S. aureus* colonies

*S. aureus* colonies were initially identified by colony morphology, with one colony from each blood agar plate confirmed as *S. aureus* using matrix-assisted laser desorption ionization time-of-flight (MALDI-TOF) mass spectrometry (VITEK MS, Biomerieux). Positively identified *S. aureus* colonies were further assessed for antibiotic resistance and susceptibility profiles for penicillin, erythromycin, flucloxacillin, co-trimoxazole, and doxycycline. Briefly, multiple suspensions were made for each isolate according to Macfarland standards, then incubated with a dried disc of each antibiotic. Growth rates and minimum inhibitory concentrations were determined using the VITEK2 (Biomerieux). Flucloxacillin susceptibility of each isolate was further assessed using a cefoxitin disc on Mueller Hinton agar, incubated in aerobic conditions at 35 °C. To evaluate intrapersonal strain-level variability, up to 20 *S. aureus* colonies were collected and sub-cultured on nutrient agar slopes for three CRS and three disease control patients. For subsequent patients, up to five *S. aureus* isolates were selected for subculture.

The culture slopes were transported to the University of Auckland, New Zealand and stored at 4 °C for up to 48 h until each subsample was re-streaked onto MSA plates. These agar plates were incubated overnight at 5% CO_2_, 37 °C. A single colony was sub-cultured in sterile tryptic soy broth (TSB) for 4 h at 37 °C and 200 rpm. Each broth culture was subsampled and stored at − 80 °C in either RNA*later* or a 1:1 volume of 80% glycerol for downstream analyses.

### Lysostaphin DNA extraction and *spa*-typing of *S. aureus* isolates

At the end of the sample collection period, *S. aureus* from − 80 °C glycerol stocks were grown on MSA plates for *spa*-typing. A single colony from each isolate was placed into 100 µL of PCR-grade water, and gently vortexed at 3000 rpm for 10 s. To lyse cells, 1 µL of 1 mg/mL lysostaphin (Sigma-Aldrich) was added to the vortexed colony and pipetted to mix, before incubation in a 37 °C water bath for 30 min. Lysostaphin was inactivated with a 99 °C incubation for 10 min in a thermomixer. After cooling, samples were centrifuged at 13,000 rpm for 2 min to pellet cell debris.

Each *spa* gene PCR reaction contained 10 × Buffer, 10 mM MgCl_2_, 2.5 mM dNTPs, 0.2 µL HotStar DNA Polymerase (Qiagen), 10 µM of each primer *spa* mod5 F (5′ TAA-AGA-CGA-TCC-TTC-RGT-GAG-C 3′) and *spa* R (5′ CAG-CAG-TAG-TGC-CGT-TTG-CTT 3′), PCR-grade water, and 2 µL template DNA for final volume of 50 µL. Thermocycler conditions for *spa* gene amplification were as follows: 15 min initial denaturation at 95 °C, followed by 30 cycles of 94 °C for 1 min, 56 °C for 45 s, and 72 °C for 1 min, and a final extension step at 72 °C for 7 min. PCR-grade water was used as a negative control and genomic *S. aureus* Newman strain DNA as a positive control for each PCR reaction.

PCR amplicons and controls were visualised on a 1% agarose gel (w/v) containing SYBR Safe DNA Gel Stain (Invitrogen Co., Carlsbad, USA) run in 0.5 × TBE buffer at 90 V for 45 min. Yield (ng/μL) and purity (260/280 nm absorbance ratio) of amplicons were determined spectrophotometrically using the NanoDrop ND-1000 (NanoDrop Technologies Inc., Wilmington, USA). A total of 160 amplicons from 10 CRS patients and 8 disease controls were sent to Macrogen, Inc. (Seoul, South Korea) for purification and Sanger sequencing. *spa*-type assignment of amplicon sequences was carried out using Ridom Staphtype software v2.2.1 (Ridom Bioinformatics software, GmbH, Münster, Germany). All 160 amplicons were confidently assigned a *spa*-type. Due to the small number of *spa*-types, they could not be positively clustered into clonal complexes.

### Bacterial 16S rRNA gene amplicon sequencing

A subset of the swab samples (13/18) collected from the middle meatus of patients that cultured positive for *S. aureus* in this study were included in other studies (Table S2)^8,55–59^. For those samples where corresponding bacterial 16S rRNA gene amplicon sequences were not already available, DNA was extracted, bacterial 16S rRNA gene V3V4 region amplified, and purified as previously described^56^. Purified amplicons were submitted to Auckland Genomics for library preparation using a dual-indexing approach with Nextera technology and 2 × 300 bp paired-end sequencing using Illumina MiSeq.

### Bioinformatic sequence processing

Raw sequencing data were processed following the DADA2 pipeline version 1.16 in R statistical program version 3.6.1^60^. Sequences utilising 2 × 300 bp paired-end sequencing were trimmed during quality filtering so the same sized insert fragment across all data were analysed. For all data, primers were trimmed, the maximum number of expected errors was set as maxEE = 2 and the maximum number of ‘N’ bases set to maxN = 0. All other parameters were as recommended. ASVs were generated after merging of forward and reverse reads for each dataset. The ASV sequence tables generated from each sequence type (2 × 250 bp paired-end and 2 × 300 bp paired-end) were combined using the ‘mergeSequenceTables’ command. To account for dataset-specific differences generated during quality filtering and merging, any sequences that were identical up to shifts or length variation were collapsed together using the ‘collapseNoMismatch’ command. Merged amplicons < 300 and > 452 bp length were excluded, then chimeric sequences removed. ASVs were assigned taxonomy up to species level using the expanded Human Oral Microbiome Database RefSeq version 15.2 which includes full length 16S rRNA gene sequences^61^.

Previous studies suggest no significant differences exist between the left and right sinuses in terms of bacterial composition^62^. Therefore, data from left and right middle meatuses were combined according to sums of ASV counts. ASVs < 0.01% overall were removed and data were rarefied to 16,339 sequence counts per sample. This final ASV table contained 260 ASVs across all 18 subjects. The alpha diversity metrics Observed richness, Shannon diversity and Simpson diversity were calculated in ‘phyloseq’ version 1.28.0. Alpha diversity metrics and taxa plots were visualised using ‘ggplot2’ version 3.3.2^66^. Beta diversity was assessed through calculating homogeneity of dispersion within groups followed by ‘adonis’ for partitioning of variation in the model in ‘vegan’ version 2.5-6.

### Statistics

Carriage prevalence of *S. aureus* and demographic statistical analyses of CRS and disease control patients were assessed using R statistical program version 3.6.1.

Differential abundance analyses of taxonomy-assigned ASVs between CRS and control group bacterial community sequence data were conducted using the R package ‘DESeq2’^63^. All default parameters were used in DESeq standard analysis, including “BH” multiple pairwise comparison *p*-value adjustment. Significant correlations between the relative abundances of bacteria were assessed using the package ‘corrplot’ in R^67^. Briefly, ASVs < 1% of overall abundance were filtered, retaining 18 ASVs which were assessed using Spearman correlations. *P*-values with “BH” multiple pairwise comparison corrections were calculated from the correlation matrix. The correlation matrix was visualised using ‘ggplot2’. These results were used to guide co-cultivation assays. All statistical data interpreted significant results as *p* < 0.05.

### *D. pigrum* and *S. aureus* co-culture assay

The *D. pigrum* species used for co-culture assays was isolated from the middle meatus of a disease control subject. This subject was not culture positive for *S. aureus* at the time of sample collection. The bacterium was initially identified by colony morphology then MALDI-TOF as described above. Co-culture assays were conducted as previously described with a few modifications^24^. *S. aureus* and *D. pigrum* cells were grown in brain heart infusion (BHI) broth at 37 °C and 200 rpm. Each inoculum was diluted in BHI broth to an OD600 of 0.5 ± 0.05 for all experiments. For experiments evaluating the effect of *S. aureus* on established *D. pigrum*, 10 µL spots of *D. pigrum* were inoculated on BHI agar medium and incubated for 2 days. After 2 days, 10 µL spots of each *S. aureus* strain were inoculated adjacent to the pre-grown *D. pigrum*. For experiments evaluating the effect of *D. pigrum* on established *S. aureus*, 10 µL spots of *S. aureus* were inoculated on BHI agar medium and incubated overnight. The next day, 10 µL spots of *D. pigrum* were inoculated adjacent to each pre-grown *S. aureus* strain. All co-culture assays were conducted in triplicate and compared with positive controls of each strain and negative control BHI plates that had no bacterial inoculum. Inhibition was assessed daily and photographically documented using the Synbiosis Protocol3 (Cambridge, UK).

## Supplementary Information

Supplementary Figures.

Supplementary Tables.

## Data Availability

Bacterial 16S rRNA sequence data not previously published can be found at NCBI under the accession SUB8696473.
