# Supplementary material for: Characterising clinical Staphylococcus aureus isolates from the sinuses of patients with chronic rhinosinusitis
Source: Sci Rep. 2021 Nov 9;11:21940. doi: 10.1038/s41598-021-01297-0 (PMC8578559; doi:10.1038/s41598-021-01297-0)
Supplement: Supplementary file 1 — Supplementary Figures. [file 41598_2021_1297_MOESM1_ESM.pdf]

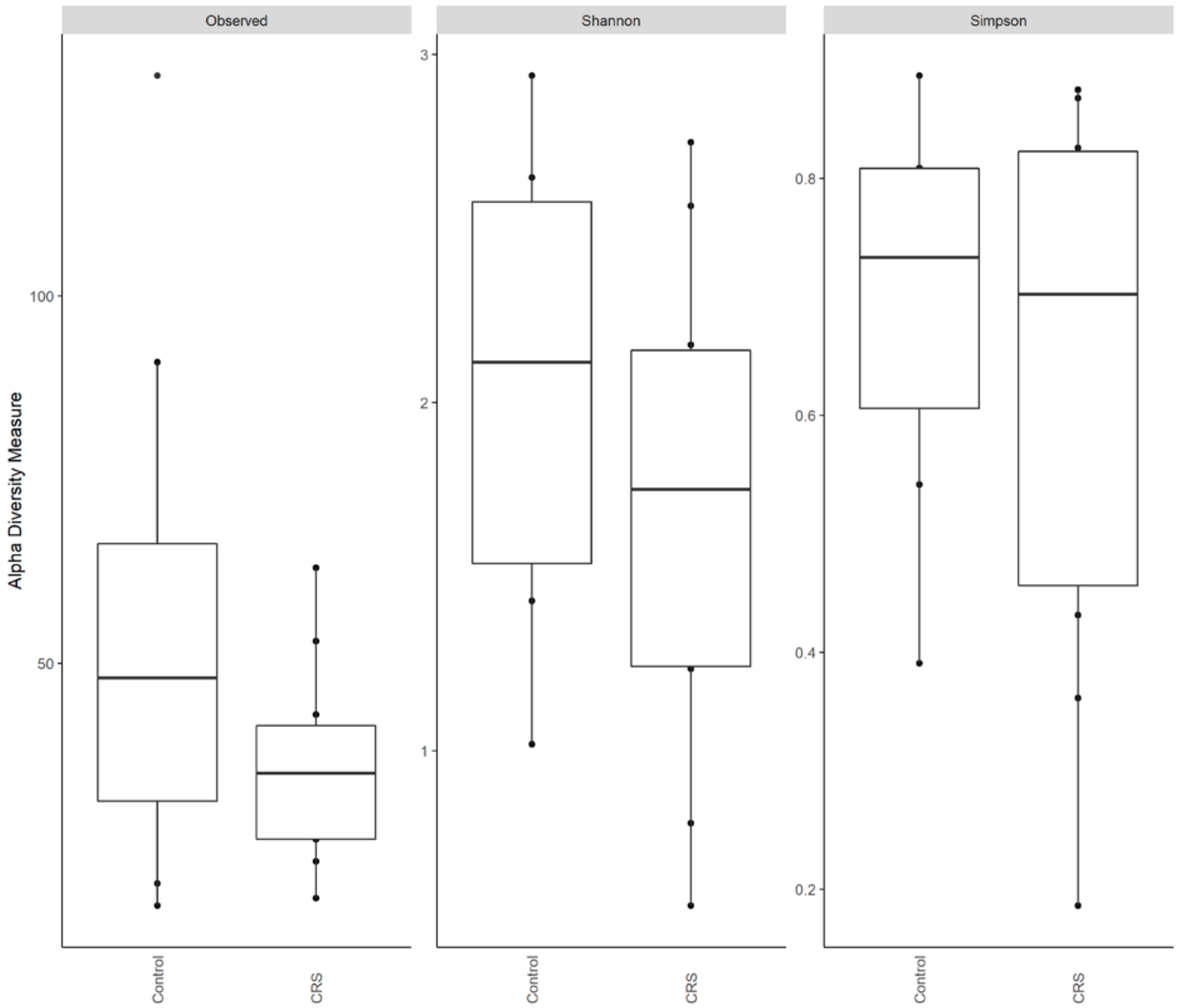

Figure S1. Alpha diversity metric boxplots comparing observed amplicon sequence variant (ASV) richness, Shannon diversity and Simpson results between disease control and chronic rhinosinusitis (CRS) patients. Median values are indicated by the line within each box, and the box extends to the upper and lower quartile values. Outlier data points are indicated by closed circles.

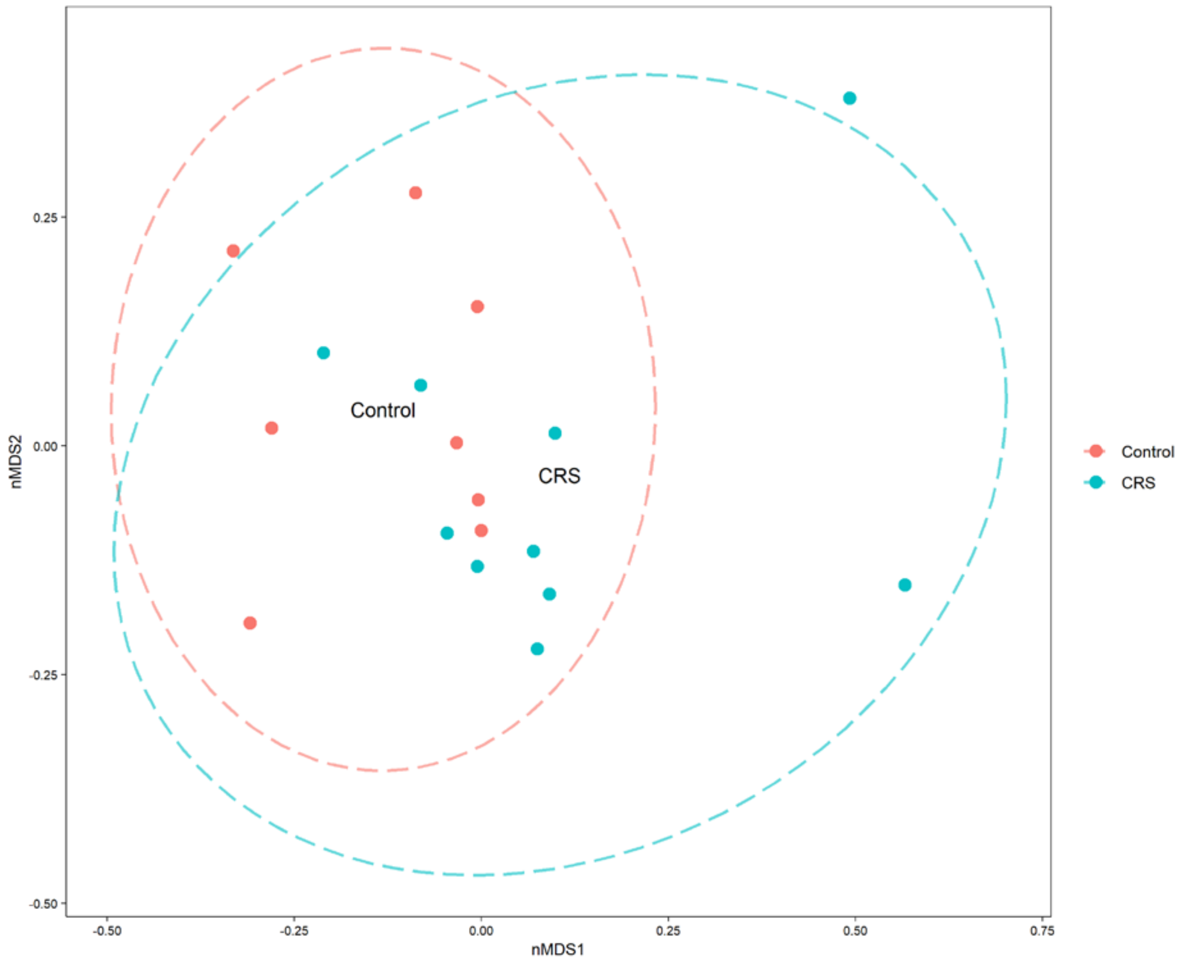

Figure S2. Bray-Curtis dissimilarity non-metric multi-dimensional scaling (NMDS) plot of control and chronic rhinosinusitis (CRS) bacterial community composition from middle meatus sinuses. The sinus bacterial community from each of the 18 patients is shown. The ellipses represent the mean of the description coordinates at the center, and the dispersion of the ellipses were calculated using the standard error of the weighted average of the covariance matrix group scores.
