# Supplementary material for: Characterising clinical Staphylococcus aureus isolates from the sinuses of patients with chronic rhinosinusitis
Source: Sci Rep. 2021 Nov 9;11:21940. doi: 10.1038/s41598-021-01297-0 (PMC8578559; doi:10.1038/s41598-021-01297-0)
Supplement: Supplementary file 2 — Supplementary Tables. [file 41598_2021_1297_MOESM2_ESM.pdf]

Supplementary Tables

Table S1. Patient demographic and clinical characteristics including antimicrobial prescriptions prior to surgery and medication history. CRS = chronic rhinosinusitis, NZE = New Zealand European. [1]LM = Lund-Mackay radiological score. Antibiotics prior to surgery refers to within 4 weeks of surgery. Medication history refers to medication within the 12 months before surgery.

Subject number	Patient	Diagnosis	Sex	Age at surgery	Ethnicity	Smoker	LM total[1]	Polyposis	Comorbidities	Antibiotics prior to surgery	Medication history
S02	RCTC17	CRS	M	49	NZE	No	16	No	Hypertension	Amoxicillin Trihydrate APO-AMOXI 500mg Capsules 22	Roxithromycin ARROW-ROXITHROMYCIN 150mg Tablets 28, Fluticasone propionate FLIXONASE 50mcg Nasal Spray 120, Doxycycline hydrochloride DOXINE 100 100mg Tablets 56, Doxycycline hydrochloride DOXINE 100 100mg Tablets 56
S03	RCTC19	CRS	M	21	NZE	No	14	No	No	Nil	
S04	RCTC4	CRS	M	60	NZE	No	11	Yes	No	Nil	Fluticasone propionate FLIXONASE 50mcg Nasal Spray 720, PrednisonePrednisone APO-PREDNISONE 5mg Tablets 42, Fluticasone propionate FLIXONASE 50mcg Nasal Spray 120, Amoxicillin 500mg, Potassium clavulanate 125mgAmoxicillin 500mg, Potassium clavulanate... AUGMENTIN 500mg/125mg Tablet 21
S05	S379	CRS	M	59	NZE	Ex	17	No	No	Nil	
S06	S382	CRS	M	55	NZE	No	15	No	No	Amoxicillin 500mg, Potassium clavulanate... AUGMENTIN 500mg/125mg Tablet 30	Doxycycline hydrochloride DOXINE 100 100mg Tablets 20, Budesonide BUTACORT AQUEOUS 100mcg/dose Nasal Spray 200, Amoxicillin 500mg, Potassium clavulanate 125mgAmoxicillin 500mg, Potassium clavulanate... AUGMENTIN 500mg/125mg Tablet 84, Prednisone APO-PREDNISONE 20mg Tablets 21, Beclomethasone dipropionate ALANASE 100mcg/dose Nasal Spray 200
S07	S411	CRS	M	24	NZE	No	14	No	Asthma	Amoxicillin 500mg, Potassium clavulanate... AUGMENTIN 500mg/125mg Tablet 21	Amoxicillin 500mg, Potassium clavulanate... AUGMENTIN 500mg/125mg Tablet 21
S08	S415	CRS	F	51	NZE	No	15	No	Asthma	Nil	Fluticasone propionate FLIXONASE 50mcg Nasal Spray 240, Fluticasone propionate FLIXONASE 50mcg Nasal Spray 240, Prednisone APO-PREDNISONE 5mg Tablets 84, Doxycycline hydrochloride DOXINE 100 100m, Amoxicillin 500mg, Potassium clavulanate... CURAM DUO 500mg/125mg Tablet 42
S09	S417	CRS	F	22	Pacific_Island	No	20	Yes	Asthma	Doxycycline hydrochloride DOXINE 100 100m	Amoxicillin 500mg, Potassium clavulanate... CURAM DUO 500mg/125mg Tablet 42 , Fluticasone propionate FLIXONASE 50mcg Nasal Spray 240, Fluticasone propionate FLIXONASE 50mcg Nasal Spray 240, Prednisone APO-PREDNISONE 5mg Tablets 84
S13	S473	CRS	M	38	NZE	No	18	Yes	Cystic Fibrosis	Ciprofloxacin CIPFLOX 750 750mg Tablets 28 /	Prednisone APO-PREDNISONE 5mg Tablets 90, Azithromycin APO-AZITHROMYCIN 250mg Tablets 12, Azithromycin APO-AZITHROMYCIN 250mg Tablets 12, Ciprofloxacin CIPFLOX 750 750mg Tablets 28, Prednisone

											APO-PREDNISONE 5mg Tablets 90, Ciprofloxacin CIPFLOX 750 750mg Tablets 28, Azithromycin APO-AZITHROMYCIN 250mg Tablets 12, Co-trimoxazole TRISUL 480mg Tablets 26, Azithromycin APO-AZITHROMYCIN 250mg Tablets 12, Ciprofloxacin CIPFLOX 750 750mg Tablets 28, Ciprofloxacin CIPFLOX 750 750mg Tablets 28, Doxycycline hydrochloride DOXINE 100 100mg Tablets 20, Budesonide BUTACORT AQUEOUS 100mcg/dose Nasal Spray 200, Roxithromycin ARROW-ROXITHROMYCIN 150mg Tablets 60, Prednisone APO-PREDNISONE 5mg Tablets 84, Doxycycline hydrochloride DOXINE 100, Budesonide BUTACORT AQUEOUS 100mcg/dose Nasal Spray 200, Doxycycline hydrochloride DOXINE 100 100mg Tablets 20
S14	S486	CRS	M	58	NZE	No	24	Yes	Reflux	Nil	
S17	S383	Control	M	42	NZE	Yes	0	No	Asthma	Doxycycline hydrochloride DOXINE 100 100mg Tablets 14	Doxycycline hydrochloride DOXINE 100 100mg Tablets 14, Roxithromycin ARROW-ROXITHROMYCIN 300mg Tablets 30 , Prednisone APO-PREDNISONE 5mg Tablets 84, Prednisone APO-PREDNISONE 5mg Tablets 84, Fluticasone propionate FLIXONASE 50mcg Nasal Spray 360, Fluticasone propionate FLIXONASE 50mcg Nasal Spray 360
S18	S384	Control	F	45	NZE	Ex	0	No	Hypertension	Roxithromycin ARROW-ROXITHROMYCIN 150mg Tablets 60	Roxithromycin ARROW-ROXITHROMYCIN 150mg Tablets 28, PrednisonePrednisone APO-PREDNISONE 20mg Tablets 14, PrednisonePrednisone APO-PREDNISONE 20mg Tablets 14, Fluticasone propionate FLIXONASE 50mcg Nasal Spray 120
S10	S419	Control	M	38	Asian	No	0	No	No	Roxithromycin ARROW-ROXITHROMYCIN 150mg Tablets 20	Ciprofloxacin CIPFLOX 500 500mg Tablets 14, Ciprofloxacin CIPFLOX 500mg Tablets 14 , Prednisone APO-PREDNISONE 20mg Tablets 30, Prednisone APO-PREDNISONE 20mg Tablets 6, Prednisone APO-PREDNISONE 5mg Tablets 32
S11	S420	Control	M	63	NZE	No	0	No	No	Amoxicillin 500mg, Potassium clavulanate. CURAM DUO 500mg/125mg Tablet 21	Prednisone APO-PREDNISONE 20mg Tablets 20, Prednisone APO-PREDNISONE 5mg Tablets 70, Doxycycline hydrochloride DOXINE 100 100mg Tablets 28
S12	S429	Control	F	28	NZE	No	0	No	Asthma	Amoxicillin trihydrate ALPHAMOX 500mg 500mg Capsules 30	Budesonide 200mcg + Eformoterol 6mcg SYMBICORT 200/6 200/6 Dry Powder Inhaler 120
S15	S490	Control	F	46	NZE	No	0	No	No	Nil	
S16	S495	Control	F	45	NZE	No	0	No	No	Amoxicillin Trihydrate APO-AMOXI 500mg Capsules 21	
S01	S475	Control	F	30	NZE	No	0	No	Graves’ Disease	Doxycycline hydrochloride DOXINE 100 100mg Tablets 20	Budesonide BUTACORT AQUEOUS 100mcg/dose Nasal Spray 200, Roxithromycin ARROW-ROXITHROMYCIN 150mg Tablets 60, Fluticasone propionate FLIXONASE 50mcg Nasal Spray 120, Fluticasone propionate FLIXONASE 50mcg Nasal Spray 360

Table S2. Samples sequenced for bacterial community composition analyses in this study, and their accession data if already published as part of an existing study. LMM = left middle meatus, RMM = right middle meatus, CRS = chronic rhinosinusitis.

Subject	Sample	accession	Side	Citation	Diagnosis
S02	RCTC17	PRJNA638969	LMM	Lux et al 2020	CRS
		SUB8696473	RMM		
S03	RCTC19	PRJNA638969	LMM	Lux et al 2020	CRS
		SUB8696473	RMM		
S04	RCTC4	PRJNA638969	LMM	Lux et al 2020	CRS
		SUB8696473	RMM		
S05	S379	By request	LMM + RMM single file	Wagner Mackenzie et al 2018	CRS
S06	S382	By request	LMM + RMM single file	Wagner Mackenzie et al 2018	CRS
S17	S383	SRP092370	LMM	Hoggard et al 2016	Disease
		SRP092370	RMM	Hoggard et al 2016	control
S18	S384	SRP092370	LMM	Hoggard et al 2016	Disease
		SRP092370	RMM	Hoggard et al 2016	control
S07	S411	SUB8696473	LMM		CRS
S08	S415	SUB8696473	LMM		CRS
S09	S417	SUB8696473	LMM		CRS
		SUB8696473	RMM		
S10	S419	PRJNA390854	LMM	Biswas et al 2017	Disease
		PRJNA390854	RMM	Biswas et al 2017	control
S11	S420	PRJNA390854	LMM	Biswas et al 2017	Disease
		PRJNA390854	RMM	Biswas et al 2017	control
S12	S429	PRJNA390854	LMM	Biswas et al 2017	Disease
		PRJNA390854	RMM	Biswas et al 2017	control
S13	S473	PRJNA639382	LMM	Lux et al 2020	CRS
		SUB8696473	RMM		
S14	S486	PRJNA639382	LMM	Lux et al 2020	CRS
		SUB8696473	RMM		
S15	S490	PRJNA639382	LMM	Lux et al 2020	Disease
		SUB8696473	RMM		control

S16	S495	SUB8696473<br>SUB8696473	LMM<br>RMM		Disease control
S01	S475	SUB5004386<br>SUB5004386	LMM<br>RMM	Wagner Mackenzie et al 2019	Disease control
